# Engaging diverse underserved communities to bridge the mammography divide

**DOI:** 10.1186/1471-2458-11-47

**Published:** 2011-01-21

**Authors:** Kimberly K Engelman, Ana Paula Cupertino, Christine M Daley, Trish Long, Angelia Cully, Matthew S Mayo, Edward F Ellerbeck, Mugur V Geana, Allen Greiner

**Affiliations:** 1 Department of Preventive Medicine and Public Health, University of Kansas Medical Center, Kansas City, KS, USA; 2 University of Kansas Cancer Center, Kansas City, KS, USA; 3 Center for American Indian Community Health, University of Kansas Medical Center, Kansas City, KS, USA; 4 Department of Biostatistics & Informatics, University of Kansas Medical Center, Kansas City, KS, USA; 5 Department of Internal Medicine, University of Kansas Medical Center, Kansas City, KS, USA; 6 School of Journalism and Mass Communications, University of Kansas, Lawrence, KS, USA; 7 Department of Family Medicine, University of Kansas Medical Center, Kansas City, KS, USA

## Abstract

**Background:**

Breast cancer screening continues to be underutilized by the population in general, but is particularly underutilized by traditionally underserved minority populations. Two of the most at risk female minority groups are American Indians/Alaska Natives (AI/AN) and Latinas. American Indian women have the poorest recorded 5-year cancer survival rates of any ethnic group while breast cancer is the number one cause of cancer mortality among Latina women. Breast cancer screening rates for both minority groups are near or at the lowest among all racial/ethnic groups. As with other health screening behaviors, women may intend to get a mammogram but their intentions may not result in initiation or follow through of the examination process. An accumulating body of research, however, demonstrates the efficacy of developing 'implementation intentions' that define when, where, and how a specific behavior will be performed. The formulation of intended steps in addition to addressing potential barriers to test completion can increase a person's self-efficacy, operationalize and strengthen their intention to act, and close gaps between behavioral intention and completion. To date, an evaluation of the formulation of implementation intentions for breast cancer screening has not been conducted with minority populations.

**Methods/Design:**

In the proposed program, community health workers will meet with rural-dwelling Latina and American Indian women one-on-one to educate them about breast cancer and screening and guide them through a computerized and culturally tailored "implementation intentions" program, called *Healthy Living Kansas - Breast Health*, to promote breast cancer screening utilization. We will target Latina and AI/AN women from two distinct rural Kansas communities. Women attending community events will be invited by CHWs to participate and be randomized to either a mammography "implementation intentions" (**MI**^**2**^) intervention or a comparison general breast cancer prevention informational intervention (**C**). CHWs will be armed with notebook computers loaded with our Healthy Living Kansas - Breast Health program and guide their peers through the program. Women in the **MI**^**2 **^condition will receive assistance with operationalizing their screening intentions and identifying and addressing their stated screening barriers with the goal of guiding them toward accessing screening services near their community. Outcomes will be evaluated at 120-days post randomization via self-report and will include mammography utilization status, barriers, and movement along a behavioral stages of readiness to screen model.

**Discussion:**

This highly innovative project will be guided and initiated by AI/AN and Latina community members and will test the practical application of emerging behavioral theory among minority persons living in rural communities.

**Trial Registration:**

ClinicalTrials (NCT): NCT01267110

## Background

Breast cancer is the most common malignancy among Hispanic women in the United States and is the leading cause of death from cancer [1]. Breast cancer is the second leading cause of cancer death among AI/AN women [2]. Breast cancer-specific 5-year survival rates for AI/AN women (84.0%) and Latinas (85.8%) are among the lowest of any racial ethnic group in the United States. Only African American and Other Pacific Islander women have lower 5-year survival rates [3]. While breast cancer mortality rates have steadily fallen for most racial/ethnic groups, mortality rates have increased among AI/AN women [4]. Hispanic/Latina women are more likely to be diagnosed at a later stage than non-Hispanic white women [5]. During 2000-2003, 54% of Hispanic women were diagnosed with local stage cancer compared to 63% of non-Hispanic white women [1]. AI/AN women were also more likely than Whites to be diagnosed with late stage disease [6]. Diagnostic stage disparities are most likely secondary to under utilization of routine mammography.

While not all breast cancers are identified by mammography, it is the most effective method for detecting cancer at the earliest most treatable stage [7]. Results of randomized trials and population-based screening evaluations have demonstrated that regular screening mammography can reduce breast cancer mortality [7]. Unfortunately, many minority and underserved women remain unscreened or are screened infrequently and thus are at risk for late stage at diagnosis and for increased mortality from breast cancer. In fact, AI/AN and Latina women have consistently had the lowest screening rates of all racial/ethnic groups [7].

### Barriers to Mammogram Utilization

A number of factors play a role in keeping mammography utilization at less than optimal levels. Numerous barriers have been identified that impede women from completing breast cancer screening. As with other health screening behaviors, women may intend to get a mammogram but their intentions may not result in initiation or follow through of the examination process [8], due, in part, to getting hung up on one or more barriers inherent in the mammography scheduling, examination, and follow-up process. Studies conducted with AI/AN women and Latinas have found similar barriers to those of other racial and ethnic groups. Screening barriers among Latinas and AI/AN women include fear of pain [9,10]; beliefs [9,10]; and embarrassment [9,11]; and lack of health insurance [9-12]. Mack et al. report that "lack of affordability was the most frequently cited perceived barrier to screening among Latinas overdue for screening" [13]. More than twice as many Hispanics report no usual source of care than do white non-Hispanics, 35% versus 15% [14]. Transportation and cost and taking time off from work [11] also influence the screening decisions of Latinas [13]. Women with less than a high school education, who have no health insurance coverage, and who are recent immigrants, are least likely to have had a recent mammogram [15]. Language is an additional barrier for many Latinas [9] and some AI/AN women [16].

Daley et al. of our research team conducted focus groups with AI community leaders and providers in Northeast Kansas. This study found that mammography cost and transportation were key among the reported barriers to screening. AI/AN women without health insurance must pay out of pocket for mammograms if the Indian Health Service clinic where they receive care does not offer mammograms. Focus group participants recommended culturally tailored written and oral materials containing specific information about how and where to get a mammogram as a means to improve utilization among AI/AN women. They also recommended outreach to culturally relevant events and locations.

### Access to Mammography

Access issues that may be classified as barriers to mammography include limited hours of mammography facility operation, acceptance of self-referrals, ease of making an appointment, waiting room time, the use of reminder postcards or telephone calls, and location/type of facility. For the AI/AN population, this may also entail the pervasive unavailability of Indian Health Service practitioners and facilities. For example, all enrolled members of federally-recognized tribes are eligible for free health services at Indian Health Service (IHS) facilities and most AI/AN receive at least some of their care at IHS facilities. The IHS has 12 area offices overseeing 550 healthcare facilities across the US. The IHS does not have funding to place mammography machines in all clinics or to transport women to clinics that have them [17]. As such, many women go outside the IHS for breast cancer screening.

The IHS Holton Service Unit in Northeast Kansas is comprised of one IHS health center (White Cloud Health Station) and two tribal health centers (Kickapoo Health Center and Prairie Band Potawatomi Family Health Center). Four American Indian Nations (Kickapoo; Iowa Tribe of Kansas and Nebraska; Prairie Band Potawatomi; Sac and Fox) reside within this region. The Holton Service Unit area office is staffed by a variety of medical professionals including physicians, family nurse practitioners, nurses, a pharmacist, and diabetic health educators. The Holton Service Unit serves a five county area (Brown, Jackson, Doniphan, Jefferson, and Pottawatomie).

The Four Tribes Women's Wellness Program, at the Prairie Band Potawatomi Family Health Center, provides outreach services for the Kansas Early Detection Works program. Women without health insurance or who are underinsured ($2500 unmet deductable), and who are 50-64 years of age, are eligible for free breast and cervical cancer screening and are given a voucher to receive a mammogram at no charge. The Kansas Department of Health and Environment also enrolls women 40-49, meeting the same criteria, in a parallel program funded by the Susan G. Komen for the Cure Foundation.

In Garden City, KS, women enrolled in the Early Detection Works program are screened for cervical cancer and receive a clinical breast exam at one of two locations: the Finney County Health Department or the United Methodist Mexican-American Ministries clinic. Latinas enrolled in the Early Detection Works program or the Susan G. Komen for the Cure program, are given a mammogram voucher. The Health Department's bi-lingual outreach worker helps women schedule a mammogram at a local hospital. Many of the Latinas in Garden City are employed by one of the two meat packing plants located in or near Garden City. While some of these women have health insurance through their employers, they face transportation barriers and may be reluctant, or unable, to take time-off to obtain routine screening.

### Avenues to Reach the Rural Kansas Latino and AI Communities

A recent study highlights the role of community programs for outreach to poor and minority women and recommends careful consideration of community based and other approaches outside of the traditional purview of medical care to encourage use of mammography among hard to reach women [18]. Community and church-based events may be perfect venues for reaching and educating underserved minority women who have limited access to mammography [19]. In fact, community health fairs nationwide have grown to become an integral part of reaching Latinos to provide education, screening, and referral to community health resources. Community health fairs fill critical healthcare needs in many Latino communities. In addition to these efforts, academic and religious institutions, philanthropic and social service agencies in communities across the country have joined to support health fairs to serve Latino communities by reaching the underserved and connecting individuals to community clinics and related health resources.

### Rationale for Framing the Project Within the Precaution Adoption Process Model (PAPM) and Incorporating the Concept of Implementation Intentions into an Intervention to Promote Breast Cancer Screening

The PAPM and the concept of "implementation intentions" form the basis for our intervention approach [20,21]. Factors addressed by the intervention will be individualized based on computerized assessments that document a PAPM stage, collect information on barriers to and perceptions around breast cancer screening and, for the **MI**^**2 **^intervention arm participants, identify each participant's implementation intentions for scheduling, getting to and receiving a mammogram.

The PAPM was chosen for this study because it includes specific stages for "unaware" and actively "deciding" individuals. "Awareness" and some degree of breast cancer screening knowledge may be a necessary precursor to behavioral interventions intending to advance screening adherence [22]. Recent studies of tailored health communications and behavioral counseling have shown that among individuals with no knowledge of a given area of health, interventions have little effect on promoting related healthy behaviors [23,24]. For breast cancer screening, "awareness" and knowledge deficits may be an especially salient issue among our two targeted minority populations (Latinas and American Indian/Native Alaskans).

Although prior breast cancer screening interventions have delivered programs to encourage participants to move from intentions to action by emphasizing the importance of breast cancer screening, more may be necessary to help participants identify and specifically agree to the intermediate action steps leading to completion of screening. For those in a less advanced PAPM stage ("Unaware," "Unengaged," and "Deciding"), this might involve identifying and agreeing to contact health care and peer information sources to learn more about the pros and cons of screening. For those individuals who have decided to be screened, this might involve stating exactly when and who the patient will contact to set up a screening test.

Our model assumes that, for many rural dwelling Latina and AI/AN women, much needs to be done to move from intent to be screened (motivation) to actual breast cancer screening action and follow through (volition). Emerging behavioral theory offers improved theoretical constructs for addressing this issue. For example, theoretical elaborations of the Theory of Planned Behavior [25,26] and the Theory of Reasoned Action [27,28], which emphasize the need to consider "implementation intentions," have garnered empirical support across a variety of behavioral outcomes, including smoking cessation, condom use and sexual behavior, and cancer screening [29,30]. This emerging body of research highlights the advantages of encouraging target groups to develop tailored action plans that specify the "when", "where" and "how" the intention will be activated.

It is believed that there are two stages--motivational and volitional--through which people pass before performing a behavior [31,32]. The motivational stage involves one's orientation toward engaging in the behavior and culminates in the formation of a behavioral intention. The volitional stage culminates in the actual performance of the behavior in question. Thus, whereas models such as the Theory of Planned Behavior [25] provide clear guidance as to what motivates people (e.g., behavioral intentions), they are less clear about how motivation is translated into action. Gollwitzer's [29] concept of implementation intentions is important in this regard. Implementation intentions are volitional strategies that can be used to ensure that behavioral intentions are translated into action. Whereas behavioral intentions (e.g., "I plan to have a mammogram in the next three months") summarize one's motivation to engage in behavior, implementation intentions are plans that specify the conditions under which a target behavior will be performed (e.g., "I will schedule a mammogram for Friday, September 16^th ^by calling the scheduling clerk at XYZ mammography facility"). As described initially by Gollwitzer and colleagues [29], implementation intentions characterize a process of strategic automaticity whereby the initiation of goal-directed responses becomes more automatic and is possible without as much direct conscious intent. Intervention strategies using implementation intentions have been successful in clinically linked settings. For example, Sheeran and Orbell [30] showed that an implementation intention intervention increased attendance at cervical cancer screening by more than 20% compared with a control group. Implementation intentions have also been shown to increase vitamin supplement intake [33], breast self-examination [34], vigorous physical activity [35], and functional ability after surgery [36].

**Figure 1:**
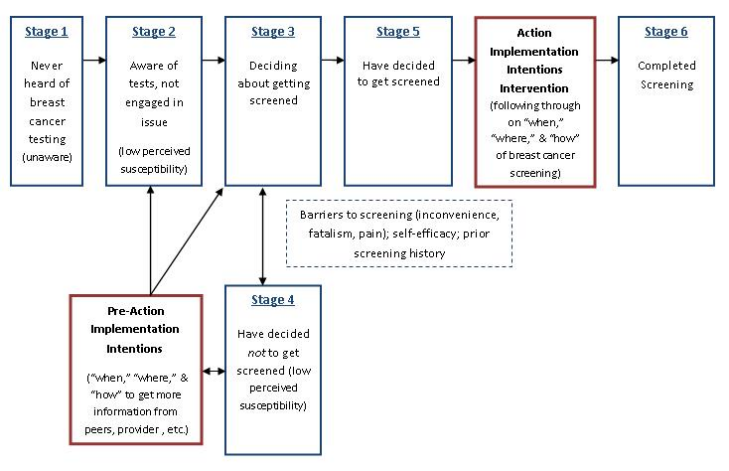
**The Integrated Precaution Adoption Process Model and Implementation Intentions Concept Applied to Breast Cancer Screening**.

As with the PAPM, implementation intentions have been shown most applicable for deliberate or one-time actions. For the proposed study we see implementation intentions as having primary salience within the have "decided to get screened" stage of the PAPM, but they also can have information seeking applicability at the "unaware," "unengaged," "deciding," and "decided not to" stages (see Figure 1). Implementation intentions provide a useful mechanism for helping individuals think through a plan to handle barriers interfering between a decision to act and the completion of the action (if near an Action stage) or seek more information (if they are near a Pre-action stage). Judging from prior studies which have shown a robust effect with "implementation intentions" interventions [21,30,37-39], participant's statements of the specific "how," "when," and "where" they will complete breast cancer screening should sharply enhance examination completion, even when the process may be considered "Inconvenient", "invasive", "painful" or "unpleasant."

#### Conceptual Model: Applying the Precaution-Adoption Process Model and Implementation Intentions to Breast Cancer Screening

The proposed research is grounded within the framework of the Precaution Adoption Process Model (PAPM) [40,41] and the concept of 'implementation intentions' [21,30,42]. Variables and concepts essential for tailoring implementation intentions along the PAPM stages are shown inside stage boxes in parentheses in Figure 1. The Precaution Adoption Process Model posits that individuals are staged along a behavioral continuum. Participants with no knowledge of breast cancer screening would be considered to be in **stage 1 **- "unaware." In a pilot cancer screening study completed by our Healthy Living Kansas research team, the vast majority of participants were able to move beyond the 'unaware' stage in our pilot projects after a brief educational session. At the end of session, 95% of participants stated they intended to complete colon cancer screening and accepted FOBT kits. Subsequently, only 22% of those accepting cards sent them in prior to a reminder call. This suggests that many individuals will report that they have "decided to be screened" but many may actually be in a "deciding" stage. Our intervention, as outlined in Figure 1, will be tailored to "Action Implementation Intentions" for those who have "decided to be screened" (**stage 5**) and "Pre-Action Implementation Intentions" for those who are "unaware" or have low perceived breast cancer susceptibility and need to seek information resources (health care provider, peers, etc.). We believe this "unengaged" (**stage 2**), still "deciding" (**stage 3**), or has "decided not to be screened" (**stage 4**) cohort will be significantly smaller than the have "decided to be screened" group.

As described above in the Background section, the Precaution Adoption Process Model (PAPM), developed by Weinstein and colleagues, is a stage theory of health behavior [40]. Stage theories attempt to define the process of behavior change and the series of steps required to fulfill behavior change. The PAPM describes the adoption of new preventive behaviors (precautions) and cessation of health-risk behaviors. The PAPM addresses an individual's awareness and the junctures along the decision-making process. Once an action decision is reached there still may be specific barriers to completing a specified action. It is best suited to behaviors with deliberate action rather than habitual patterns that develop gradually over time [43].

The PAPM has been applied to health behaviors including mammography [44], osteoporosis prevention [45] and management [46], and home radon testing [47]. While other stage-based health behavior theories have been applied to cancer screening [48,49], use of the PAPM with the implementation intentions concept is novel. Like other screening tests for colon, cervical, and prostate cancer, many women, including minorities, demonstrate a lack of awareness and/or knowledge about breast cancer and screening. Therefore, a model such as the PAPM that addresses people who are unaware of their risk or their screening options is appropriate.

In general, health behavior theories assume a certain level of knowledge about the health issue in question. After all, people need to know something to form perceptions and attitudes about the behaviors that lead to or prevent disease. Lack of awareness of the issue defines the PAPM's first stage. In the second stage, people become aware but are not yet at a point where they are actively considering the precautionary behavior. An individual in this stage lacks engagement due to inadequate personal relevance. Perceived susceptibility is presumed to be important at this stage. If, and when, people become engaged in the issue, they progress to a decision-making phase (Stage 3). Self-efficacy, barriers, and any prior experience with the screening test might be important in this decisional stage. Decision-making can result in a decision not to act (Stage 4) or a decision to act (Stage 5). However, deciding to act is not the final step in the process. It is at the decisional point that new barriers or low self-efficacy can again become apparent and need to be addressed directly. We propose addressing these issues through an application of the "implementation intentions" construct and have labeled this Action Implementation Intentions in the gap between decision and test completion. Our prior work [50] and other studies [8] have shown that even once a decision has been made and individuals are motivated to complete cancer screening, many individuals still fail to follow through with volitional features of testing procedures. This gap between decision and action is not unique to the PAPM [25,51].

The development of plans to overcome specific logistic or perceptual barriers can facilitate the translation of intent into action. For breast cancer, the decision to get screened may be separate from the decision of where to go to obtain screening services. Multiple steps are clearly involved in following through with test scheduling, completion, and follow-up. Our proposed project will explore and define these constructs as they apply to breast cancer prevention; the culturally sensitive intervention will test the model and its assumptions, and a 120-day post randomization follow-up survey will confirm participant progression through PAPM stages, determine changes in reported breast cancer screening barriers, and evaluate response to implementation intentions among minority women.

This study was approved by the University of Kansas Medical Center Human Subjects Review Committee.

## Study Aims And Hypotheses

### Aim #1

To compare the 120-day post randomization mammography screening completion rates of individuals who receive a computerized mammography "implementation intentions" (**MI**^**2**^) intervention, versus a comparison computerized condition of general breast cancer prevention health education (**C**).

#### Hypothesis

Not up-to-date or never screened individuals who receive a mammography "implementation intentions" intervention (**MI**^**2**^) will be significantly more likely to complete a screening mammography test at 120 days post randomization than those receiving a general breast cancer prevention health education intervention (**C**).

### Aim #2

To compare the screening mammography self-reported barriers after 120-days post randomization between those receiving a mammography "implementation intentions" intervention (**MI**^**2**^) and those receiving only a general breast cancer prevention health education intervention (**C**).

#### Hypothesis

Those who receive the **MI**^**2 **^intervention will report fewer barriers to screening mammography completion than those receiving a general breast cancer prevention health education intervention (**C**).

### Aim #3

To compare changes in mammography screening Precaution Adoption Process Model decisional stage from baseline to 120 days post randomization between individuals in the **MI**^**2 **^and **C **study arms.

#### Hypothesis

Individuals who receive the **MI**^**2 **^intervention will show significantly more movement in PAPM stage at 120 days post randomization than those receiving a general breast cancer prevention health education intervention (**C**).

## Methods/Design

### Study 1: Development - Formative Research

This study is a randomized intervention testing the efficacy of a novel intervention program for increasing breast cancer screening use among rural minority women. The study employs a partnership intervention between Community Health Workers and Latina and American Indian women to provide breast cancer education and promote the use of an 'implementation intentions-based' approach for breast cancer screening (See Table 1: Implementation Plan). Eligible (i.e., 40 years of age or older and not up-to-date on breast cancer screening), consenting participants will complete a baseline computerized Healthy Living Kansas-Breast Health (HLK-BH) assessment while attending one of many community or church-based events scheduled within their respective home community. The HLK-BH program will gather data on past breast cancer screening behavior, breast cancer screening barriers, perceived breast cancer susceptibility and screening self-efficacy. Consenting age-eligible, not up-to-date participants will be randomized to a mammography "implementation intentions" intervention arm (**MI**^**2**^) or a control (**C**) arm (described in detail below). Participants will be enrolled from well attended community events scheduled on an annual basis in and around Mayetta in northeast Kansas (to capture the American Indian population) and Garden City in southeast Kansas (an area highly populated with Latinos). Details regarding site selection, randomization, intervention methods, sample size calculations, and data analysis procedures are described below. We project that 121 women 40+ years old and not up-to-date with mammography screening will be required for each of the study arms to detect the proposed intervention effects.

**Table 1:** Implementation Plan

Study	Tasks	Year
**Study 1**: Development and Formative Research	Refine computer program, refine assessments (e.g., HLK-Breast Health, 120-day post randomization telephone follow-up), convene community advisory board, pilot test full HLK-Breast Health computer program.	1 - 2

**Study 2**: Randomized Trial	Finalize recruitment procedures, implement HLK-Breast Health program and 120-day post randomization telephone follow-up, perform preliminary data cleaning and initial data analysis.	3 - 5

**Study 3**: Data Analysis and Dissemination	Complete data cleaning, data analysis, project-related manuscripts, and follow-up grant development.	4 - 5

#### Design

This study will be a randomized trial that will evaluate the differential impact of the **MI**^**2 **^(intervention) and **C **(control) study arms on 120-day post randomization breast cancer screening utilization rates. Randomization will occur at the participant level. Women will be randomized to a treatment group within each community outreach recruitment event. This stratified random sampling design will minimize the variation between the **MI**^**2 **^and **C **intervention arms by allocating the variation between each Northeast and Southwest Kansas recruitment event to the strata parameters. In doing so, the variation between interventions will be minimized, much like a randomized complete block design.

#### Healthy Living Kansas-Breast Health Computer Program Development

##### Computer Program

Notebook computer will be used to conduct the Healthy Living Kansas-Breast Health (HLK-BH) program. Notebook software interfaces will be programmed by personnel from the University of Kansas (KU) Design for Health group - a network of communications, information technology, and web design professionals that have collaborated with our Healthy Living Kansas research team over the past several years. We anticipate that the HLK-BH program will be conducted on an electronic computer platform but will ask Latina and American Indian community members to decide whether they would prefer an Internet-enabled or notebook PC-based program (where data are saved on the computer and not uploaded to the Internet). For individuals reticent with completing the HLK-BH program via any electronic format, we will have a paper and pencil version available as well. The KU Design for Health staff will utilize their graphic design and effective communication campaign knowledge to ensure that the final HLK-BH product has a crisp, user-friendly look to it while still being easily navigable. With the input of community members, we will work to make the program culturally sensitive by having two programs with identical data elements but one tailored to Latinas and the other tailored to American Indian women. Each program will also be enhanced with clickable voice and video clips in English and Spanish to assist participants with lower literacy levels. Program team members will work to achieve a notebook product that has high literary contrast, readable font type and functional font size with an overall presentation that is complete with compatible non-glaring hues and tones.

##### Healthy Living Kansas Breast Health Assessment (HLK-BH)

Study investigators have collaborated to develop a comprehensive cancer assessment for use in our current Healthy Living project. The breast cancer questions contained in the previously developed cancer assessment will provide the platform from which the research team will develop the HLK-BH program for the notebook computers used in the proposed study.

#### HLK-BH Part I

Part I of the HLK-BH program will begin by screening for eligible study participants. Eligible participants will be 40-74 years of age and not up-to-date on breast cancer screening as defined by the American Cancer Society. Persons who are 40-74 years of age or older but who report being up-to-date on breast cancer screening will receive a one-page on-screen summary report that indicates they are up-to-date on breast cancer screening and reinforces their healthy breast behavior. Persons deemed eligible (i.e., meet age and breast cancer screening criteria) will continue with Part II of the HLK-BH program.

#### HLK-BH Part II

Part II of the HLK-BH program will begin with an on-screen consent procedure. The research team currently uses an on-screen consent procedure in our Healthy Living project (described in the Preliminary Studies section). If a participant does not consent to the study, she will immediately receive an on-screen summary report that promotes breast cancer screening, and describes why being up-to-date with breast cancer screening is important. Consenting participants proceed with answering questions about breast cancer screening awareness, breast cancer testing preferences, breast cancer screening barriers, perceived breast cancer susceptibility, screening self-efficacy, and breast cancer risk status. Demographic questions will assess marital status, employment status, years of education, income, race, and ethnicity.

Whenever possible, HLK-BH questions will be adapted from already existing and validated questions from widely used surveys (e.g., Behavioral Risk Factor Surveillance System Survey, National Health Interview Survey). Members of the Community Advisory Boards will review all components of the HLK-BH and notebook computer presentation to identify ways in which it may be improved. Previous studies conducted by the research team using computer tablets and tablets in primary care practices revealed the importance of keeping assessments brief. The research team will strive to restrict the assessment to 10-20 minutes. All HLK-BH questions will be subjected to computer readability analyses. We will use computer software developed by Readability Calculations that assesses text readability using nine recognized formulae [52].

The full HLK-BH tablet program will be pilot tested with community members to ensure that the program length is manageable and is running smoothly. Persons who participate in the HLK-BH notebook computer pilot will not participate in the proposed randomized trial. The research team will work with the advisory boards and community members to develop additional methods to assure assessment brevity.

#### Community Health Worker Training

CHWs will be trained and meet compliance criteria prior to recruiting study participants. Training elements will include: 1) reviewing the implementation intentions protocol manual, 2) reading a training workbook with a series of publications and reviews of breast cancer screening guidelines, breast cancer screening intervention studies, implementation intentions theory, and Health Education theory, 3) participating in training sessions conducted by study investigators, 4) conducting simulated recruitment intervention sessions, with sessions recorded and supervised by the P.I., 5) participating in follow-up training sessions conducted by study investigators, and 7) participating in ongoing evaluation by the P.I..

Training content will include the theoretical models and key principles underlying implementation intentions and the intervention overview. In addition, the study P.I. will train CHWs to respond to participant concerns regarding breast cancer screening and interaction with the health care system (e.g., exam initiation, procedural details, follow-up). CHWs will be trained to educate participants about approaching their physician or ancillary staff to request a mammogram referral, how to address concerns with exam procedures and, when to refer participants to their physician for questions and concerns related to breast cancer screening. To ensure CHWs are adequately trained, they will conduct practice sessions with each other, with volunteer participants, and with pilot participants prior to recruiting participants for the study. These sessions will be videotaped and the P.I. and Project Director will conduct supervisory sessions to provide guidance and feedback to the CHWs.

For purposes of CHW training and ensuring fidelity to principles and protocols of the implementation intentions, we will use a rating form adapted from our recent Healthy Living Kansas colon cancer screening study. Adherence to CHW principles will be rated on a 1-7 (Poor/Never to Excellent/Always) scale. Scores of 4 or higher represent adherence at the level of "good" or "often". Acceptable performance will be indicated by a mean score of 5 or higher with no score on any criterion being below 3. CHWs will have to achieve acceptable performance across all criteria on three consecutive practice participants before being permitted to recruit participants. If performance drops below the threshold (i.e., a mean of five and a minimum of three on all criteria), additional supervision will be conducted. Each of the CHWs subsequent sessions will then be supervised until three consecutive tapes again meet the threshold.

### Study 2. Randomized Trial

#### Participant Eligibility

Eligibility criteria for randomized trial participation include: individuals must be 40 years of age or older and not up-to-date with breast cancer screening as defined by current American Cancer Society recommendations (see Table 2). Individuals with an acute medical illness or a history of breast cancer will be excluded from the study. Although no formal assessment of dementia or psychiatric illness will be performed, participants demonstrating impaired cognitive function or inappropriate affect or behavior will be excluded from the study. Individuals with another household member enrolled in the study also will be ineligible. Individuals who have been enrolled in the study will not be eligible for repeat enrollment. This is true even if greater than one year has passed since their initial participation. Anyone not eligible for the study but interested in breast cancer screening and information will be given printed self-help materials (*What you need to know about Breast Cancer*, National Cancer Institute, *Breast Cancer and You*, Centers for Disease Control). Participants must have a current, active mailing address and access to a functioning telephone to ensure that the 120 day telephone follow up can be completed.

**Table 2:** Participant Eligibility

Inclusion Criteria	Exclusion Criteria
• Latina or AI/AN woman residing in one of participating communities • Aged ≥40 years of age • Not up to date on mammography screening • Home address & access to a working telephone • Responded to 120-day post randomization follow-up call	• Receipt of mammogram within past year • Acute medical illness, history of breast cancer • Cognitive impairment or inappropriate affect or behavior • Another household member enrolled in the study

#### Availability of Participants

We will recruit and randomize a total of 290 participants (145 randomized to the **MI**^**2 **^arm and 145 randomized to the **C **arm). Participants will be recruited from annually scheduled and well attended community events in and around the Mayetta and Garden City, KS communities. Garden City is the Finney County seat. Approximately 50% of the county population, and Garden City's population, is non-Hispanic White while 45% of the nearly 41,000 people living in Finney County are Latino (approximately 9,000 Hispanic/Latina females). Our American Indian recruitment in Northeast Kansas will include a five county region; Brown (pop. 10,009/52% female/9% AI/AN); Jackson (pop. 13,240/50% female/7% AI/AN); Doniphan (pop. 7,753/51% female/2% AI/AN); Jefferson (pop. 18,421/50% female/1% AI/AN); and Pottawatomie (pop. 19,695/50% female/1% AI/AN). The AI/AN female population in each county is: Brown = 470; Jackson = 452; Jefferson = 100; Potawatomie = 69; and Doniphan = 58.

Annual community events are common within both target communities. Amongst other events, Mayetta and surrounding areas are home to the Prairie Band Potawatomi Pow Wow (held in June) and Prairie Band Potawatomi Health Fair (September), Kickapoo Pow Wow (July), Sac and Fox Health Fair (September), and the Ioway Health Fair (October). Garden City and its surrounding communities is home to a Cinco de Mayo celebration (May), Community Mexican Fiesta (September), Tyson Health Fair (October), Binational Health Fair (October), and several community events sponsored by the six area churches. Using three CHWs per community, we anticipate being able to recruit approximately 150 women in each community. If, upon a midcourse evaluation, we determine that recruitment is behind schedule, we will supplement our recruitment efforts by partnering directly with health clinics in our target communities to recruit within clinic waiting rooms.

#### CHW/Participant Partnership Implementation Intentions (MI^**2**^) Intervention

Each participant in the **MI**^**2 **^intervention arm will receive brief one-on-one breast cancer screening education delivered in person by a CHW. Following the education information delivery, the CHW will guide participants through the Healthy Living Kansas-Breast Health computerized screening and intervention program. This program will guide participants through a screening/eligibility assessment and implementation intentions protocol via automated computerized algorithms. If, upon screening, a person is deemed eligible to participate in the randomized trial (e.g., they are eligible for but not up to date on breast cancer screening), the computer program will automatically and immediately assign them to either the intervention or control arm. The total time required per participant to complete the education session and HLK-BH notebook computer program will be kept to a maximum of 20 minutes.

For persons who are randomized to the **MI**^**2 **^intervention arm, the HLK-BH program will guide participants through a series of implementation intentions questions to fully delineate step-by-step breast cancer screening intentions of participants (the when, where and how of screening) and encourage follow through on these intentions. Breast cancer screening information, relevant to the stated intentions of the participant will then be delivered over the remaining course of the program. Implementation intentions questions and breast cancer screening information in the HLK-BH program will be adapted with input from community and advisory board members and from questions and materials used successfully in our prior implementation intentions-based cancer screening studies. The HLK-BH program will, by necessity, consist of two primary phases designed to: 1) elicit participants' implementation intentions and 2) encourage follow through. Although implementation intention tasks will differ for each participant, a sample list of tasks is presented in Table 3. In a typical program session, participants will be asked to declare whether they "intend" to get breast cancer screening. If they are unsure or do not intend to get screened within the next 120 days, they will be taken through a series of implementation intention questions that specifically address when, where, and how they will gather more information on breast cancer screening or engage in discussions about breast cancer screening with their physician, family, or peer group. Individuals intending to receive screening will be led through a series of questions about when, where, and how they will be screened. The questions will include specifics on making contacts with the health care system, obtaining authorizations from health care providers, scheduling appointments, arranging for transportation to mammography locations, and avoiding potential behavioral pitfalls along each juncture of the aforementioned process. All implementation intention questions will be tailored to the race/ethnicity of the participant. The HLK-BH program also will provide suggestions that might help the participant overcome obstacles to test completion (inconvenience, pain, embarrassment). To assist persons with low literacy levels, the computer program will evaluate and organize data as they are entered and then deliver a tailored intervention message using video clips and narrated graphics. In this manner, the program will select messages that are tailored to race/ethnicity and gender matched to individual participants. Participants will wear headphones. The primary goal of the HLK-BH program for **MI**^**2 **^participants is to clarify implementation intentions and address barriers which might prevent follow through on these intentions.

**Table 3:** Sample Implementation Intention Task Analyses for MI^2 ^Participants

Participants "Unaware" Stage for Breast Cancer Screening	Participants "Decided/Deciding" About Breast Cancer Screening
• Identify a source for more breast cancer information • Specify a goal date by which the breast cancer information source will be contacted • Identify steps necessary to contact the breast cancer information source (e.g., if breast cancer information source is a health care provider: obtain telephone number, schedule an appointment, arrange transportation to and from the appointment if necessary) • Identify breast cancer screening questions to ask • Describe how information gathered will be saved and additional contacts who may be used as "second opinions"	• Identify health professional that will supply mammography referral, if necessary • Declare how to request a referral • Assess insurance status • Identify eligibility for Early Detection Works program • Telephone to schedule exam (e.g., who to call, number to call, information to have available to schedule exam) • Identify exam preparation tips to increase comfort • Arrange transportation to and from appointment if necessary • Arrange for child care if necessary

Immediately following completion of the HLK-BH session, participants will receive a printed "Implementation Intentions Confirmation Contract" that will serve as a final indicator of the stated and agreed to plan that the HLK-BH session produced. This contract will serve as a written reminder and reinforcement for each participant's breast cancer screening implementation intentions and will be tailored to each individual's Precaution Adoption Process Model behavioral stage of readiness. As with the notebook computer screens, the KU Design for Health unit will work with us to ensure the delivery of high quality, highly readable and usable printed contract.

#### Control Arm (**C**) Intervention

Each participant in the **C **intervention arm will receive the same brief one-on-one breast cancer screening education information delivered in person by a CHW as **MI**^**2 **^participants. Following the education information delivery, the CHW will guide participants through the Healthy Living Kansas-Breast Health computerized screening and intervention program. This computerized program will guide participants through a screening/eligibility assessment and additional breast health and screening education protocol. If, upon screening, a person is deemed eligible to participate in the randomized trial (e.g., they are eligible for but not up to date on breast cancer screening), the computer program will automatically and immediately assign them to either the intervention or control arm. The **C **intervention will also be infused with audio-narratives delivered by a non-matched clinician reader. This clinician will read textual material comparable to that provided in educational pamphlets distributed by the NCI and Centers for Disease Control. The information will cover prevalence and distribution of breast cancer, rates of mortality, the potential mortality reduction with early detection, early signs and symptoms of breast cancer, and primary preventive measures for reducing the incidence of breast cancer. A small amount of supplemental material may be provided to assure that the length of this session matches the length of the multimedia session provided to **MI**^**2 **^participants. As with **MI**^**2 **^participants, the total time required per participant to complete the education session and HLK-BH computer program will be kept to a maximum of 20 minutes.

During the control arm HLK-BH program, participants will be reminded of the importance of breast cancer screening and highlight NCI-recommended healthy breast behaviors such as regular physical activity and a diet rich in fruits and vegetables. These HLK-BH sessions will rely on a "health educator" methodology. Furthermore, the CHW will be instructed to not initiate specific behavioral advice or use behavioral counseling strategies following participant completion of the control arm HLK-BH program. If, however, a participant asks for specific behavioral advice related to the formulation of implementation intentions, the CHWs will answer the participant's question briefly and document the question and their reply.

All participants (intervention and control) will received a $25 gift card to reimburse them for their time involved with completing the HLK-BH program assessment.

#### 120-Day Post Randomization Follow-Up

**Figure 2:**
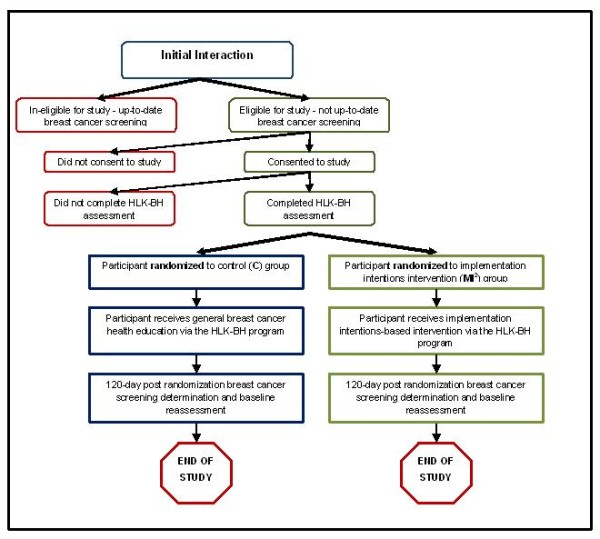
**Flow of Participants**.

All **MI**^**2 **^and **C **participants will receive a follow-up telephone call from a CHW to reassess breast cancer screening status. For example, participants will be asked if screening was performed within the last 120 days since the initial intervention, and if so, when it was performed, and when the participant plans to have their next routine breast cancer screening examination. The CHW also will re-administer portions of the HLK-BH program questions via the telephone and ask questions regarding breast cancer testing utilization, breast cancer screening barriers, perceived breast cancer susceptibility, screening self-efficacy, and breast cancer risk status and reassess Precaution Adoption Process Model behavioral breast cancer screening stage. Program evaluation and satisfaction questions will also be asked of each participant near the end of this final assessment. By re-administering portions of the HLK-BH program questions at the 120-day post randomization follow-up, the research team will be able to evaluate the differential impact of the **MI**^**2 **^intervention compared to the **C **intervention on the primary (e.g., breast cancer screening utilization) and secondary outcomes (e.g., breast cancer screening barriers and progression through the PAPM stages). Refer to Figure 2 for an overview of the flow of research participants.

#### Project Instrumentation

The purpose of all survey project instrumentation is to collect data for use in developing individually tailored materials and to allow for analysis of study main effects with appropriate covariate controls. The measured constructs should also provide information on which variables are responsible for intervention effects. All measures except for "Implementation Intentions" measures will be given to study participants prior to randomization and thus to participants in both arms (**MI**^**2 **^and **C**).

Though many of the scales and instruments selected for the study have been used in previous studies, the complete baseline survey instrument will be pilot tested with Latina and female AI/AN community members within our two target communities. Feedback from this pilot testing phase will be used to revise the instrument and to prepare it for administration. Our goal is to establish an instrument that can be completed in 10-15 minutes using a notebook computer.

#### Demographics

Data on gender, education, marital status, age, income, employment status, and race/ethnicity will be collected. With respect to race/ethnicity, study participants will be asked to identify themselves as being Black/African American (and allow for further ethnic identification as U.S. born, Caribbean immigrant, African immigrant, Jamaican, etc.), Hispanic, White, Native American/American Indian, Asian, or Other [53].

#### PAPM Stage

The model's authors provide a staging algorithm and recommended question formats for staging other health behaviors [40,54]. We will adapt the staging questions to breast cancer screening and use a six-item scale: "Have you ever heard about breast cancer screening?"; "Have you been tested for breast cancer?" and "Which of the following best describes your thoughts about getting tested for breast cancer: I've never thought about it, I'm undecided, I've decided I don't want to, I've decided I want to get tested." Necessarily, stage will be asked prior to the behavior questions so that responses are not influenced or triggered by the questions about specific screening tests.

#### Perceived Susceptibility to Breast Cancer, Perceived Screening Benefit, and Screening Barriers

We will utilize the Revised Susceptibility, Benefits, and Barriers Scale developed by Champion [55]. This 19 item scale has been used widely by researchers and has shown to be a reliable and valid assessment.

#### Self-Efficacy to Complete Mammography

A single item ("how confident are you that you can complete breast cancer screening?") [8] will be combined with a four-item scale that showed excellent internal consistency (alpha = 0.82) and factor loadings from 0.65-0.68 in a confirmatory factor analysis [56].

#### Prior Breast Cancer Screening Test Utilization

Participants will be asked if they have ever been screened for breast cancer and how long ago the test was received. This nine-item set of questions has been used in pilot studies by the research team [50] and was adapted from Behavioral Risk Factor Surveillance System questions. This set of questions will also ask whether or not a participant has undergone any breast cancer testing for diagnostic purposes.

#### Cancer Fatalism

We will employ an adapted Powe Cancer Fatalism inventory to evaluate beliefs toward early detection, treatment, and cancer myths (10 questions).

#### Implementation Intentions - only delivered to **MI**^**2 **^participants

Implementation intentions measures have been adapted from the work of Gollwitzer and Sheeran, et al [30,39]. To assess Pre-Action/Pre-contemplation Implementation Intentions, participants will be asked where, when, and how they will seek out more information on breast cancer prevention ("do you think you will try to get more information about breast cancer?" "Do you believe you will talk to any of the following individuals about breast cancer prevention?" "Can you pin down a date you think you will first talk to this person about breast cancer prevention? ___/____/______"). For Action/Contemplation Implementation Intentions, participants will be asked specific action-oriented questions regarding mammography. A participant who describes an intention to complete screening mammography may also be asked (among other things) to set a specific date they plan to be screened, whether they need to get a healthcare provider referral, what mammography facility they will call to set up their appointment (the telephone number will be supplied to participants automatically by the HLK-BH program once a facility is selected).

##### Process Evaluation

It is necessary to document and analyze the process of program implementation to verify the integrity of the intervention, allow for correction of problems as they occur, provide data to examine the relationship between program delivery and program outcomes, and to guide future investigations [57-59]. Therefore, a comprehensive set of process assessments, including both qualitative and quantitative data, will be designed or adapted from previous studies to measure implementation, participation, and fidelity.

We plan to measure characteristics of the participants and community health and environmental issues that may influence breast cancer screenings. In addition, we will collect information that may provide useful insight into refining protocols and materials. Another aim of the evaluation is to provide a general description of the context in which the intervention is conducted. Table 4 summarizes the variables to be measured and the data collection instruments to be used.

**Table 4:** Summary of Process Evaluation Measures

Source	Instruments	Variables of Interest
Participants	HLK-BH program; 120 day follow-up telephone call	Demographics, ease of use of notebook computer, type of insurance coverage (i.e., Medicare, uninsured, commercial), new/emerging illness, benefits/barriers (# of miles to screening facility), discomfort with screening procedure

Community Health Workers	CHW training session tracking form; attendance records	Attendance at training sessions, turnover, transfers, level of support for program (e.g., importance of breast cancer screenings)

External and competing programs	Indian Health Service & United Methodist Mexican American Ministries Clinic Director questionnaire	Programs/Information/Policies designed to increase breast cancer screenings not initiated by us

#### Community Participants

We will assess the ability of the participants to use the notebook computer by direct observation and follow-up with questions regarding the ease of use during our planned exit survey. In addition to demographic characteristics, we will collect type of insurance coverage, which has been found to have a differential effect on cancer screenings [60]. The Community Health Workers will perform these observations. We will also determine if screening rates could be affected by dominant mammography facilities distributed asymmetrically across our target communities [61], number of miles to screening mammography facilities, and level of perceived discomfort with screening procedure, etc. To identify issues related to perceived benefits and challenges, we will ask a subsample of participants about the helpfulness, relevance, convenience, level of satisfaction, etc. of their Healthy Living Kansas-Breast Health program Community Health Worker. Measuring participant's satisfaction and soliciting information about potential benefits and barriers will provide valuable feedback about the extent to which the activities have met with patient expectations [62]. We will use structured interviews to be completed during the end of the study to determine benefits and barriers, helpfulness of Community Health Workers, why they did or did not get screened, and whether or not they would recommend the program to others. These random assessments will be conducted via telephone for 10% of participants.

#### Community Health Workers

The quality and integrity of the HLK-BH program will be partially dependent on how the CWHs carry out the education and recruitment sessions. The ability of the CHW to conduct activities consistent with program goals will vary across workers. This variation will be related to a number of factors including training, self-efficacy, number of years experience, understanding of the overall program, and level of perceived support. We will administer a training workshop survey to the CHWs during the training session to document their attendance and their level of support for and importance of the HLK-BH screening program. Further, we will assess the CHW's level of confidence to implement the research protocol as designed. In addition, we will track turnover and/or transfer rates to document potential study personnel issues and track CHW participation in weekly group teleconference calls, encrypted data transfers, and recruitment logs. We will assess these factors by administering a questionnaire to all CHWs involved in the study.

#### HLK-BH Components

We will use a number of different strategies to document the degree to which the research protocol was implemented as planned. For example, each notebook computer or HLK-BH Internet program will track the amount of time to complete the HLK-BH assessment. We will also assess the number of individuals who were ineligible for the intervention program because they self-reported being in compliance with screening guidelines, already had a screening appointment scheduled, had an acute and/or terminal medical condition at the time of their recruitment interaction with a CHW.

#### External and Competing Programs

We will ask mammography facility personnel local to Mayetta and Garden City, KS to complete a short questionnaire to assess whether their facility received any breast cancer-related information not related to our project, which was shared with patients, or if promotional activities were conducted to increase breast cancer screenings, in addition to our research project during our study time period, which might serve to contaminate or lessen program effects. Information gained will be used to help us determine if external or competing factors might have influenced breast cancer screening objectives, posing as possible internal validity threats [63].

#### Process Evaluation Analysis

We have planned process evaluation procedures to provide detailed information regarding the intervention implementation. The process evaluation will include paper and pencil surveys, structured interviews, and monitoring logs to collect quantitative and qualitative data on specific program components and implementation issues. Monitoring the extent and quality of implementation will allow us to guard against "Type III Error" in which an intervention, not fully implemented as planned, is not a true test of the experimental hypotheses [64].

Descriptive statistics will be used to provide a general description of the context in which the intervention is being conducted (e.g., participant, CHW, and community recruitment setting characteristics). Individual characteristics, for example, may contribute to or detract from intervention effects. To monitor intervention components across recruitment communities and exposure to program elements, we will develop estimates of adherence rates from survey information and self-report measures, magnitude of intervention effects and the variability of outcome.

The content of comments from structured interviews completed by participants will be examined for common themes related to recommendations for improving training protocols and materials, and factors related to implementation of the screening program. Comments will be analyzed by employing the content analysis techniques recommended by Miles [65]. Identification of and suggestions for the removal of barriers to participation, and for procedures to enhance adherence will be summarized to allow for midcourse corrections in implementation, to refine the intervention design, and to provide direction to our intervention strategies [66].

### Study 3: Data Analysis and Dissemination

#### Data Analysis

Descriptive statistics will be generated from the sample using the demographic measures. Pearson's χ^2 ^test will be used to compare the proportions of categorical characteristics between the two intervention groups. The two-sample t-test will facilitate the comparison between the groups for characteristics measured on a continuous scale. This will allow us to evaluate the randomization, as we would expect to find roughly 5% of these findings to be statistically significant at the α = 0.05 level.

##### Aim #1

To compare the 120-day post randomization mammography screening completion rates of individuals who receive a computerized mammography "implementation intentions" (**MI**^**2**^) intervention, versus a comparison computerized condition of general breast cancer prevention health education (**C**).

For this aim, both the outcome (120-day post randomization breast cancer screening completion) and predictor (treatment group) are dichotomous, so a 2 × 2 table will be generated to compare the proportions of those completing screening between the **MI**^**2 **^versus **C **intervention groups. A χ^2 ^statistic will be used to test the null hypothesis of no difference in these proportions between these two [67]. The distribution of measured and unmeasured confounding variables (beyond community location) should be relatively balanced between the intervention groups due to the random assignment of the intervention within communities and participants. However, if serious imbalances are apparent from the descriptive statistics described above, we will use unconditional logistic regression to model this dichotomous outcome. Predictor variables will include the intervention group and the unbalanced demographic characteristics to reduce the impact of such biases. Indicator variables identifying the community will also be included to control for this stratification variable as described by Agresti [67]. The results of this logistic regression will be transformed into estimates of the proportion screened [68] adjusted for the potential confounding variables. The fit of the model will be assessed using the Deviance statistic and the Hosmer-Lemeshow goodness-of-fit test [68].

To measure the impact of attrition on the part of the study participant, a sensitivity analysis will be performed. This can be done using the methods described by Rothman and Greenland [69] by examining what the results would be under various assumed scenarios for those missing the outcome. This will include a conservative imputation approach which will assume that all of those for whom this outcome is unknown were not screened during the 120-day post randomization period.

##### Aim #2

To compare the screening mammography self-reported barriers after 120-days post randomization between those receiving a mammography "implementation intentions" intervention (**MI**^**2**^) and those receiving only a general breast cancer prevention health education intervention (**C**).

The outcome for this aim, self-reported barriers, takes integer values from zero to eleven. To analyze this outcome, we will use a two-sample t-test. If gross non-normality is present and Wilcoxon Rank Sum test will be used. In addition to testing the main effect of treatment group, the interaction between treatment arm and 120-day breast cancer screening status will also be investigated to determine whether there is a greater reduction in barriers among screeners from the **MI**^**2 **^group relative to screeners from the **C **group. In the presence of serious imbalances despite the randomization, additional predictor variables can be incorporated into this analysis using multiple regression assuming the normality assumption holds. If not, appropriate power transformations will be used on the dependent variable prior to modeling.

##### Aim #3

To compare changes in mammography screening Precaution Adoption Process Model decisional stage from baseline to 120 days post randomization between individuals in the **MI**^**2 **^and **C **study arms.

The PAPM outcome is a six-level ordinal measure and the predictor (treatment group) is dichotomous, so a 2 × 6 table will be generated. The Cochran-Armitagestatistic will be used to test the null hypothesis of no difference in the distribution of scores between these two groups versus a trend [70]. If serious imbalances are apparent from the descriptive statistics described above, we will use the cumulative logit model [67]. Predictor variables will include the intervention group and the unbalanced demographic characteristics to reduce the impact of these biases. Indicator variables identifying the community will also be included to control for this stratification variable as described by Agresti [67]. The results of this regression will be transformed into estimates of the proportion moving from one stage to the next, adjusted for the potential confounding variables. The fit of the model will be assessed using the Deviance statistic.

#### Sample Size and Sample Size Justification

This study will utilize Latina and AI/AN female participants from two distinct communities with approximately 121 randomized participants per community to test the main effects of the **MI**^**2 **^vs. **C **arms at 120 days post randomization. For this study, a total of 290 subjects will be randomized to either the **MI**^**2 **^or **C **treatment arms (to account for a possible 20% attrition rate from randomization to 120 day follow up). It is anticipated that 30% of those in the **MI**^**2 **^group will receive their breast cancer screening within the 120 day study period compared to 15% in the **C **group. A total of 242 subjects (121 per study arm) would have 80% power to detect the anticipated difference in screening rate between the **MI**^**2 **^and **C **groups at the α = 0.05 (two-sided) type I error rate (Table 5). Assuming up to a 20% attrition rate based on the research team's prior studies in rural Kansas, a total of 290 subjects will be randomized to either the **MI**^**2 **^or **C **treatment arms (145 per treatment). Additional screening rates were examined for study power (Table 5) to select an adequate sample size to detect clinically relevant differences. Numbers contained in Table 5 demonstrate that the sample will have at least 80% power to detect clinically relevant differences. Similar tables to Table 5 were also compared with varying sample sizes, with the final sample size selected being the choice that optimized both study power with the logistical constraints.

**Table 5:** Power estimates (%) for a sample size of 121 subjects per group with α = 0.05 (two-sided)

		Breast Cancer Screening - Implementation Intentions	
		25%	30%
Breast Cancer Screening	10%	87	97
		
Controls	15%	Not Relevant	80

#### Basis for projected breast cancer screening rates

In general, the literature shows that approximately 42% of Latina and 47% of American Indian/Alaskan Native women aged 40 years or older have been screened recently.

#### Basis for intervention impact estimate

A study utilizing breast cancer survivors to serve as promotoras (similar to our community health worker paradigm proposed in this study) to promote breast cancer screening resulted in a 21% uptake in screening among 141 Latina women [71]. In a separate study with Native American women, a face-to-face patient navigator breast cancer education intervention resulted in a 11% uptake in screening mammography [72]. We expect a similar screening mammography uptake rates in our proposed study.

Screening uptake data from our current Healthy Living Kansas colorectal cancer implementation intentions intervention study are not yet available. However, other implementation intention-based studies have shown positive improvements in dietary fat intake [37], vitamin supplement intake [33], breast self-examination rates [34], vigorous physical activity rates [35], and functional ability after surgery [36]. Sheeran and Orbell [30] conducted a study to investigate the impact of an implementation intention intervention on cervical cancer screening rates - a behavior similar to the primary outcome of the proposed study, breast cancer screening. Sheeran and Orbell found the implementation intention intervention to increase cervical cancer screening attendance by 23% compared with a control group. Another recent study to assess the effect of implementation intentions on testicular self examination (TSE) in men found a 25% increase in self-reported TSE vs. a no intervention group. Both studies represent substantial behavioral changes resulting from implementation intentions-based interventions. Based on these studies, we would anticipate a similar increase in breast cancer screening in a general population. However, the participants who will be randomized into one of the proposed study arms represent traditionally underserved rural minority women whose access to mammography services may be hampered. Thus, we would expect for it to be more difficult to alter their breast cancer screening behavior. Nonetheless, based on the aforementioned assumptions, we anticipate a 25% increase in CRC screening rates for **MI**^**2 **^participants versus a 10% increase in breast cancer screening rates for **C **participants.

## Discussion

In the U.S., women from Hispanic or American Indian/Alaskan Native racial/ethnic backgrounds have disproportionately low breast cancer screening rates. Later stage cancers also have been found among persons living in rural communities. Not unlike other states, Kansas has seen recent unprecedented growth of underserved rural Latino and American Indian communities. Simple 'one size fits all' programs to overcome barriers to breast cancer screening are likely not to be effective within these communities given their diverse cultures and unique mammography access issues. To be maximally effective, programs to promote breast cancer screening in these communities will need to be culturally relevant, pertinent to the specific concerns of individual community members, and provide clear direction regarding steps that advance program participants towards their intended goal. There is good empirical evidence to suggest that a program with implementation intention components (e.g., one that lays out the where, how, and when steps to screening) will be an effective method for addressing breast cancer screening underutilization. In this study, we propose to test such an 'implementation intentions' based method that is firmly seated in health behavior theory, culturally relevant, community specific, and guided and introduced by Latina and AI/AN community health workers in two distinct rural communities. The conduct of this study will move us forward in our understanding of how best to improve breast cancer screening utilization among underserved minority and rural-dwelling individuals. This type of intervention also is significant in that it is generalizable to organizations such as health plans or existing 1-800 cancer information telephone programs that have the capacity to adopt similar telephone-based counseling procedures to promote cancer screening utilization.

## Competing interests

The authors declare that they have no competing interests.

## Authors' contributions

KKE, KAG, and EFE contributed to the design of the study and identified the research questions and hypotheses. KKE and TL were responsible for obtaining ethics approval. MSM planned the statistical analyses. KKE, TL, CMD, and KAG oversee the study implementation. EFE serves as a scientific reviewer for the project. KKE, APC, CMD, KAG, and MVG are part of the study development and deployment team. AC is part of the project implementation team. All authors have read, revised, and approved the final manuscript.

## Pre-publication history

The pre-publication history for this paper can be accessed here:

http://www.biomedcentral.com/1471-2458/11/47/prepub
